# Intermittent theta burst stimulation attenuates oxidative stress and reactive astrogliosis in the streptozotocin-induced model of Alzheimer’s disease-like pathology

**DOI:** 10.3389/fnagi.2023.1161678

**Published:** 2023-05-18

**Authors:** Jelena B. Stanojevic, Milica Zeljkovic, Milorad Dragic, Ivana R. Stojanovic, Tihomir V. Ilic, Ivana D. Stevanovic, Milica B. Ninkovic

**Affiliations:** ^1^Institute for Biochemistry, Faculty of Medicine, University of Niš, Niš, Serbia; ^2^Medical Faculty of Military Medical Academy, University of Defense, Belgrade, Serbia; ^3^Laboratory for Neurobiology, Department for General Physiology and Biophysics, Faculty of Biology, University of Belgrade, Belgrade, Serbia; ^4^Institute of Medical Research, Military Medical Academy, Belgrade, Serbia

**Keywords:** Alzheimer’s disease, streptozotocin, iTBS, oxidative stress, astrocytes

## Abstract

**Introduction:**

Intracerebroventricularly (icv) injected streptozotocin (STZ) is a widely used model for sporadic Alzheimer’s disease (sAD)-like pathology, marked by oxidative stress-mediated pathological progression. Intermittent theta burst stimulation (iTBS) is a noninvasive technique for brain activity stimulation with the ability to induce long-term potentiation-like plasticity and represents a promising treatment for several neurological diseases, including AD. The present study aims to investigate the effect of the iTBS protocol on the animal model of STZ-induced sAD-like pathology in the context of antioxidant, anti-inflammatory, and anti-amyloidogenic effects in the cortex, striatum, hippocampus, and cerebellum.

**Methods:**

Male Wistar rats were divided into four experimental groups: control (icv normal saline solution), STZ (icv STZ—3 mg/kg), STZ + iTBS (STZ rats subjected to iTBS protocol), and STZ + Placebo (STZ animals subjected to placebo iTBS noise artifact). Biochemical assays and immunofluorescence microscopy were used to evaluate functional and structural changes.

**Results:**

The icv STZ administration induces oxidative stress and attenuates antioxidative capacity in all examined brain regions. iTBS treatment significantly reduced oxidative and nitrosative stress parameters. Also, iTBS decreased Aβ-_1-42_ and APP levels. The iTBS enhances antioxidative capacity reported as elevated activity of its enzymatic and non-enzymatic components. In addition, iTBS elevated BDNF expression and attenuated STZ-induced astrogliosis confirmed by decreased GFAP^+^/VIM^+^/C3^+^ cell reactivity in the hippocampus.

**Discussion:**

Our results provide experimental evidence for the beneficial effects of the applied iTBS protocol in attenuating oxidative stress, increasing antioxidant capacity and decreasing reactive astrogliosis in STZ-administrated rats.

## 1. Introduction

Alzheimer’s disease (AD) is a complex and progressive neurodegenerative disorder with no effective therapy to treat or slow down the disease progression. The main hallmark and the prevailing hypothesis postulate that the deposition of amyloid-β (Aβ) senile plaques triggers a cascade of neurotoxic events leading to neuronal and synaptic loss (Knopman et al., 2021). Initial damage triggers oxidative stress and activates glial cells which produce neuroinflammatory factors further aggravating the disease (Alzheimer's Association, 2018; Long and Holtzman, 2019). The constellation of various factors, including region-specific degeneration, leads to the well-known pathological changes such as impaired cognitive control of memory retrieval, weakening of attention, learning, spatial, and working memory reflected in impaired function of specific brain regions such as cortex, hippocampus, striatum, and cerebellum. Indeed, studies examining the regional distribution of Aβ deposits in both experimental models and AD patients showed their presence in almost all brain regions, including hippocampus, various cortical regions, cerebellum, thalamus, and amygdala (Thal et al., 2002; Whitesell et al., 2019; Tsui et al., 2022). Although the extent of Aβ deposition may vary in different brain regions, even small deposits can cause the detrimental phenotype of glial cells leading to neuronal loss (Solleiro-Villavicencio and Rivas-Arancibia, 2018). The brain is highly susceptible to oxidative damage (Bélanger et al., 2011) due to its high lipid content, high oxygen consumption, and low antioxidant capacity. Numerous studies emphasized oxidative stress as an important mediator in different stages of AD pathology (Cheignon et al., 2018; Ionescu-Tucker and Cotman, 2021; Tamagno et al., 2021). Moreover, the appearance of oxidative stress markers precedes the accumulation of visible amyloid deposits (Praticò et al., 2001; Su et al., 2008), suggesting that oxidative stress may be one of the earliest features in the AD brain (Götz et al., 1994; Zhu et al., 2007). Oxidative stress affects many metabolic pathways in the brain and causes irreversible damage to biological systems by oxidizing cells’ major biomolecules such as lipids, proteins, and nucleic acids (DNA, RNA) thus contributing to the complexity of AD pathology. For this reason, future treatment modalities should be more focused on antioxidative strategy and it seems that glia may be a promising target for modulating oxidative stress (Farbood et al., 2020).

Streptozotocin [2-deoxy-2-(3-(methyl-3-nitrosoureido)-D-glucopyranose)—STZ] is a naturally occurring chemical compound produced by *Streptomyces achromogenes*, used primarily for induction of experimental model of diabetes mellitus (Szkudelski, 2001). Much is known about the biology of both conditions (type 2 diabetes mellitus and AD-related dementias), which are parallel phenomena arising from coincidental roots in aging or synergistic diseases linked by vicious pathophysiological cycles (Arnold et al., 2018). Recently it has been reported that insulin resistance induces oxidative stress and inflammation in the brain which contributes to Aβ and tau pathology (Cai et al., 2015). However, it has been shown that intracerebroventricular (icv) injection of STZ leads to glucose hypometabolism, oxidative stress, neurodegeneration, dysfunctions in adult neurogenesis and senile plaque formation thus modeling common aspects of AD in an experimental model (Salkovic-Petrisic et al., 2013; Bassani et al., 2018). Furthermore, STZ administration affects the morphology and number of astrocytes in specific brain regions (Rostami et al., 2017; Lin et al., 2022) mimicking the changes noted in AD (Monterey et al., 2021). Also, previous studies reported that oxidative stress and amyloid pathology are associated with decreased neurogenesis in the STZ model (Sun et al., 2015), similar to one present in human AD (Ekonomou et al., 2015). It is very important to emphasize that precise mechanisms of STZ toxicity in the brain are still largely unclear. Therefore, this animal model does not reflect the underlying condition for most patients, and we should be careful when extrapolating results (Platt, 2019). However, based on the known effects of STZ, this animal model represents a good basis for examining the influence of the selected therapy on the antioxidant capacity, neuroinflammatory and amyloidogenic status of specific brain regions.

Repetitive transcranial magnetic stimulation (rTMS) is a promising approach for the treatment of different neurological diseases, including AD (Zhao et al., 2017). A protocol known as intermittent theta burst stimulation (iTBS) is a form of rTMS that requires less stimulation time, and lower intensity, and induces a long-lasting positive effect in the human cerebral cortex (Huang et al., 2005). Recent studies showed the positive effects of iTBS on cognitive performance in both AD patients (Golaszewski et al., 2021; Wu et al., 2022) and animal models (Stanojevic et al., 2022). Also, rTMS restored the disturbed balance between parameters of antioxidant protection and oxidative stress in experimental rats (Liang et al., 2021). Different TMS protocols affected the production of nuclear factor (erythroid-derived 2)-like 2 (Nrf2), 8-hydroxy-2′-deoxyguanosine (8-OHdG), Nitrite and nitrate concentration (NO_2_ + NO_3_), Superoxide dismutase (SOD), glutathione peroxidase (GPx), reduced glutathione, catalase (CAT), suggesting a therapeutic effect of TMS could be mediated at least partly by its effects on anti-oxidant/pro-oxidant mediators (for review please see Medina-Fernández et al., 2018). Furthermore, astrocytes, the important players in brain recovery and homeostasis, could mediate some of the effects of iTBS, as they can directly or indirectly respond to electrical activity (Cullen and Young, 2016). So, the focus of this research was to investigate the antioxidative impact of two sets of five-days long iTBS stimulation in the context of examining regional brain changes and to expand the potential of this animal model, as well as to get a broader insight into the mechanism and treatment of AD pathology. To our knowledge, there is no information on the antioxidant and anti-amyloidogenic effects of this specific iTBS protocol in an animal model of AD.

## 2. Materials and methods

### 2.1. Ethical statement

All experimental procedures were performed according to the decision No. 323-07-08358/2020-05 obtained from the Ethics Committee for Animal Welfare and the Ministry of Agriculture, forestry, and water economy of the Republic of Serbia, which respects the rules of the European Parliament’s Directive 2010/63/EU on the protection of animals used for scientific purposes.

### 2.2. Animals

Experiments were conducted on 10 weeks old male Wistar rats (average weight ~ 300 g). The experimental animals were housed under standardized housing conditions (temperature of 23°C ± 2°C, relative humidity of 55% ± 3%, and 12-h shift of the *light*–*dark cycle*) in polyethylene cages with free access to standard laboratory pellet food and tap water.

### 2.3. Experimental groups and time scale

**Figure 1:**
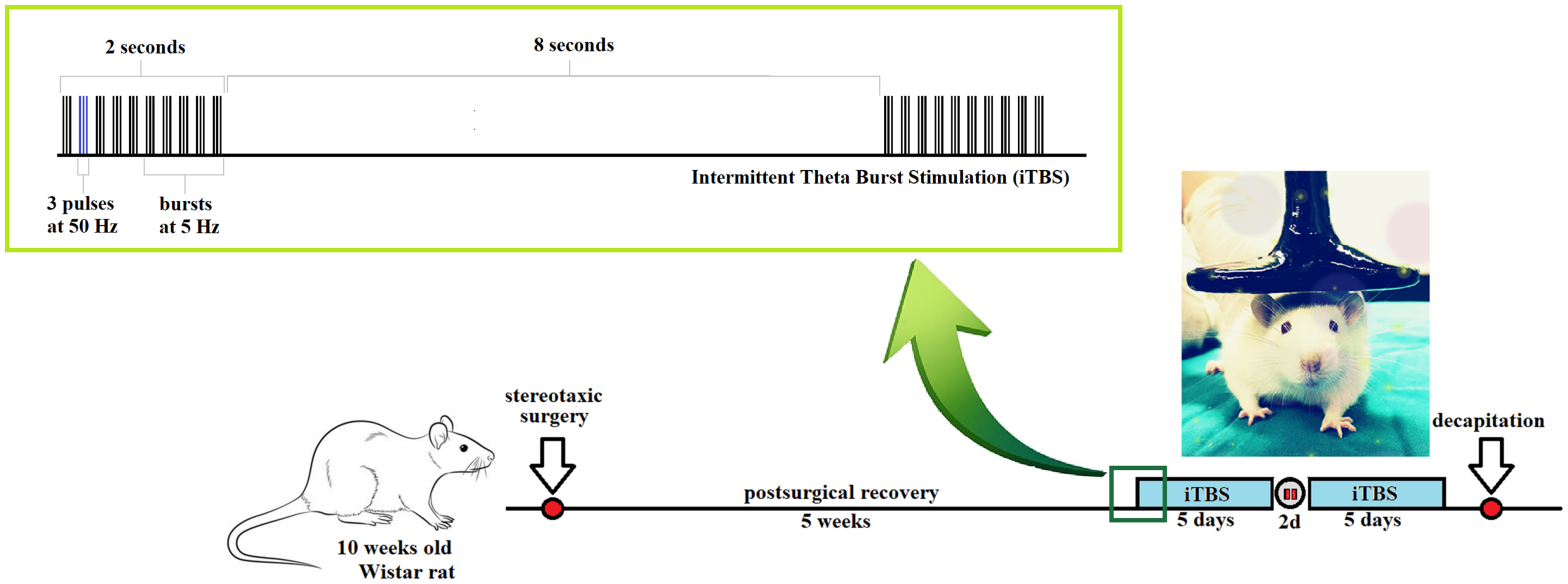
Schematic presentation of the experimental time scale: intermittent Theta Burst Stimulation (iTBS) treatment of streptozotocin (STZ)-induced Alzheimer-like disease rat model.

Animals (*n* = 35) were assigned randomly into four groups: (1) Control group (*n* = 9)—rats underwent stereotaxic surgery and received icv saline solution; (2) Streptozotocin (STZ) group (*n* = 9)—received icv STZ; (3) STZ + iTBS group (*n* = 9)—STZ rats subjected to iTBS protocol, and (4) STZ + Placebo group (*n* = 8)—STZ animals subjected to placebo iTBS noise artifact. After iTBS treatment, the decapitation was done using *Harvard Apparatus*. The scheme of the experimental design is depicted in Figure 1.

### 2.4. Stereotaxic surgical procedure

The animals underwent stereotaxic surgical procedures as previously explained (Stanojevic et al., 2022). Briefly, all animals were anesthetized with an intraperitoneal injection (ip) of Ketamidor (50 mg/kg body weight (bw); Richter Pharma AG, Austria) and Xylased 2% (5 mg/kg bw; Bioveta, Czech Republic). To prevent cerebral edema, rats received Dexaveto ip (2.5 mg/kg bw, VMD NV Belgium). Next, each animal was fixed in a laboratory stereotaxic frame (Stoetling Co., United States). The bregma was defined according to the stereotaxic coordinate atlas (Paxinos and Watson, 2007) and used as a reference point for coordinates (AP = −0.8; ML = ±1.5; DV = −3.8). Injections were carried out with an infusion pump (Harvard Apparatus, Holliston, MA, United States) using a 50 μL Hamilton microsyringe at a constant flow rate of 0.4 μL/min. Streptozotocin solution (3 mg/kg; Tocris Bioscience, United Kingdom) freshly dissolved in saline solution (Moreira-Silva et al., 2019; 0.9% NaCl; Hemofarm, Serbia) was injected icv bilaterally, in the volume of 5 μL in each ventricle. The STZ dose used for icv injection was selected based on the literature (Mehla et al., 2013; Ghosh et al., 2020) and was standardized in our laboratory. The Control group underwent the same surgical procedures but received icv of saline solution (Sirwi et al., 2021). Postoperatively, to prevent pain, infection, and dehydration, rats received *Butorphanol* (50 μg/kg bw; Richter Pharma, Austria), *Enroxil* (10 mg/kg bw; Krka, Slovenia), and 3 mL of 0.9% sodium chloride solution (Vicente et al., 2020).

### 2.5. Theta burst stimulation protocol

Theta burst stimulation (TBS) was applied in the form of iTBS, applying the protocol according to Huang et al. (2007), using a MagStim Rapid2 device and a 25-mm figure-of-eight coil (The MagStim Company, Whitland, Dyfed, United Kingdom) as previously described (Stanojevic et al., 2022). Briefly, the iTBS block was consisting of 20 trains of 10 bursts (3 pulses at a frequency of 50 Hz), repeated at 5 Hz (with 10 s intervals between trains; total duration 192 s; schematically presented in Figure 1), so each block contained a total of 600 pulses. Stimulation intensity was just below a motor threshold value, i.e., 33% of the maximum stimulator output. The motor threshold value was defined as a stimulus intensity that induces a minimal visible contraction of the upper limbs of animals stimulated with TMS. Intermittent TBS protocol treatment was applied once a day, in a form of two five-day long sessions with a two-day pause in between. During the application of iTBS, rats were manually immobilized, and the coil was gently held in direct physical contact with the animal head, positioned with the center of the coil right above the bregma. The STZ + Placebo group of animals underwent the same manual manipulation and noise artifacts exposure to iTBS protocol.

### 2.6. Homogenates preparation and total protein determination

After decapitation, the brains were carefully removed from the skull. Selected structures (prefrontal cortex, hippocampus, striatum, and cerebellum) were dissected on the ice and transferred in cryotubes into liquid nitrogen for instant freezing. Frozen brain regions were immersed in homogenization buffer (0.32 M sucrose and 5 mM TRIS, pH 7.4), homogenized, and centrifuged for 15 min at 3,000 × g, at 4°C. The supernatant was carefully collected, and the protein concentration was determined by the modified Lowry method (Markwell et al., 1978).

### 2.7. Biochemical analysis

Superoxide anion (O_2_^•−^) was determined by the reduction of nitroblue-tetrazolium—NBT (Merck, Darmstadt, Germany) in an alkaline nitrogen-saturated medium (Auclair and Voisin, 1985). Kinetic was performed at 550 nm on Ultrospec 2000 spectrophotometer. The results were expressed as nmol red NBT/min/mg protein.

Lipid peroxidation was determined spectrophotometrically by using the thiobarbituric acid reactive species (TBARS) assay, as described by Girotti et al. (1991). Two molecules of TBARS reagent (15% trichloroacetic acid +0.375% thiobarbituric acid +0.25 mol/L HCl) react with MDA, forming a complex with absorbance measurable at 492 and 650 nm. The results expressed as nmol of malondialdehyde (MDA) per milligram of proteins (mmol MDA/mg protein).

Nitrosative stress was assessed based on the nitrite and nitrate concentration. Nitrite and nitrate concentration (NO_2_ + NO_3_) was determined spectrophotometrically at 492 nm. Nitrites were assayed directly using the Griess colorimetric method (1.5% sulfanilamide in 1 M HCl plus 0.15% N-(1-naphthyl) ethylenediamine dihydrochloride in distilled water). However, nitrates were formerly converted into nitrites by cadmium reduction (Navarro-Gonzalvez et al., 1998). The concentrations of nitrites in the medium were evaluated from the standard curve generated with known nitrite concentrations and expressed as μmol/mg protein.

Total Superoxide dismutase activity (tSOD), which combines the activity of mitochondrial MnSOD and cytoplasmatic CuZnSOD, was determined using spectrophotometric measurement of a decrease in the rate of spontaneous epinephrine autooxidation at 480 nm. The kinetic activity was followed in 50 mM carbonate buffer (pH 10.2) with 1 mM EDTA after the addition of 10 mM epinephrine and 5 mM KCN for MnSOD (Sun and Zigman, 1978). CuZnSOD activity was determined as a difference between tSOD and MnSOD. The activity was expressed as units per milligram of total protein (U/mg protein). One unit is described as the amount of enzyme required for a 50% inhibitor of epinephrine autooxidation.

Catalase (CAT) activity was determined spectrophotometrically by using ammonium molybdate to produce a yellow complex with H_2_O_2_ (Góth, 1991). Kinetic was performed at 405 nm on Ultrospec 2000 spectrophotometer. CAT activity was defined as mmol H_2_O_2_ reduced per minute (mmol H_2_O_2_/min), expressed as U/mg protein.

Intracellular levels of total Glutathione (GSH) were spectrophotometrically measured at 412 nm, for 6 min, by 5,5-dithiobis-2-nitrobenzoic acid (DTNB)—oxidized glutathione (GSSG) recyclable method. The level of produced 5-thio-2-nitrobenzoic acid (TNB) was proportional to the total GSH concentration (Stohs et al., 1986). The content of total GSH was expressed as nmol GSH/mg protein.

Tissue homogenate concentration of total sulfhydryl (SH) groups was measured spectrophotometrically at 412 nm in phosphate buffer (0.2 mol + 2 mmol EDTA, pH 9) using 5,5-dithiobis-2-nitrobenzoic acid (DTNB, 0.01 M; Ellman, 1959). The results are expressed as nmol SH/mg protein.

Nuclear factor erythroid-derived 2-like 2 (Nrf2) levels in the cortex, hippocampus, striatum, and cerebellum were measured using a rat ELISA kit purchased from Fine Biotech (Wuhan, China), according to the manufacturer’s instructions (Catalog number: ER0666). Results are presented as pg/mg protein for Nrf2.

8-Hydroxy-2′-deoxyguanosine (8OHdG) concentrations in the cortex, hippocampus, striatum, and cerebellum were measured using a competitive ELISA kit purchased from Elabscience (Wuhan, China), trailing to the manufacturer’s instructions (Catalog number: E-EL-0028). Results are presented as ng/mg protein for 8OHdG.

Early growth response protein 1 (EGR1) levels in the cortex, hippocampus, striatum, and cerebellum were measured using a rat ELISA kit purchased from Fine Biotech (Wuhan, China). The procedures were performed according to the manufacturer’s Guidelines (Catalog number: ER0916). Results are presented as pg/mg protein for EGR1.

*β*-amyloid_1-42_ and Amyloid-beta precursor protein (APP) concentrations in the cortex, hippocampus, striatum, and cerebellum were measured using a rat ELISA kit purchased from Elabscience (Wuhan, China), performed according to the manufacturer’s Guidelines (Catalog numbers: E-EL-R1402 and E-EL-R2490, respectively). Results are presented as pg/mg protein for *β*-amyloid_1-42_ and as ng/mg protein for APP.

Brain-derived neurotrophic factor (BDNF) concentrations in the cortex, hippocampus, striatum, and cerebellum were measured using rat ELISA kit purchased from Fine Biotech (Wuhan, China), performed according to the manufacturer’s instructions (Catalog number: ER0008). Results are presented as pg/mg protein for BDNF.

### 2.8. Brain tissue preparation and immunohistochemical staining

The brains were carefully removed on ice, fixed in 4% paraformaldehyde (PFA) in 0.1 M PBS (pH 7.4, 24 h at 4°C), cryoprotected and dehydrated in graded sucrose (10%–30% in 0.2 M phosphate buffer; 4°C) and sectioned on the cryotome (Leica CM1850, Germany), as described before (Stanojevic et al., 2022). Afterward, 25 μm thick coronal cryosections of the dorsal hippocampus, prefrontal cortex (PFC), and caudoputamen (CPu) were collected serially, mounted on superfrost glass slides, air-dried for 2 h at room temperature (RT), and stored at −20°C until further use.

Slides were held on RT for 30 min and washed in PBS (3X). After washing, sections were blocked with 5% normal donkey serum (Abcam) at RT for an hour. Then, sections were probed with appropriate primary antibodies (Table 1) in a humid chamber at 4°C overnight. After this, sections were washed in PBS (3X) and then incubated with appropriate fluorescence dye-labeled secondary antibodies (Table 1) for 2 h at RT in a humid chamber. After washing in PBS (3X) in the dark room, the sections were mounted with Mowiol (Calbiochem, La Jolla, CA). For triple immunofluorescence staining, primary and secondary antibodies were applied separately for each labeling. Sections were analyzed by a confocal laser-scanning microscope (LSM 510, Carl Zeiss GmbH, Jena, Germany), using Ar multiline (457, 478, 488, and 514 nm), HeNe (543 nm), and HeNe (643 nm) lasers using 40X objectives and monochrome camera AxioCam ICm1 camera (Carl Zeiss GmbH, Germany). Images were captured on 40X magnification.

**Table 1:** List of antibodies.

Antibody	Source and type	Used dilution	Manufacturer
Glial fibrillary acidic protein—GFAP	Rabbit, polyclonal	1:500	DAKO, Agilent Z0334, RRID: AB_10013382
Vimentin—VIM	Mouse, polyclonal	1:300	DAKO. M0725, RRID: AB_10013485
C3	Goat, polyclonal	1:300	Thermo Fisher Scientific PA I-29715, RRID: AB_2066730
Anti-beta amyloid antibody	Rabbit, monoclonal	1:7,000	Abcam, ab201060; RRID:2818982
Anti-rabbit IgG Alexa Fluor 555	Donkey, polyclonal	1:400	Invitrogen A-21206, RRID: AB_141708
Anti-mouse IgG Alexa Fluor 647	Donkey, polyclonal	1:400	Thermo Fisher Scientific, A31571, RRID: AB_162542
Anti-goat IgG Alexa Fluor 488	Donkey, polyclonal	1:400	Invitrogen A-11055, RRID: AB_142672
Goat anti-rabbit IgG, HRP—conjugated	Goat, polyclonal	1:30,000	Abcam, ab6721; RRID: AB_955,447

### 2.9. Dot blot analysis

The samples (10 μg of proteins) were mixed with the 6× Laemmli sample buffer (375 mM Tris–HCl, pH 6.8, 12% SDS, 60% w/v glycerol, and 0.03% bromophenol blue). For detection of amyloid-β in the hippocampus, supernatants were spotted on the activated PVDF support membrane (Immobilon-P transfer membrane, Millipore, Merck, Germany) through a vacuum-based Minifold dot blot apparatus (Schleicher & Schuell Inc., Keene, N.H.) for 30 min. The membranes were blocked in 5% milk for 1 h at RT and probed with rabbit anti-rat amyloid-β antibody (Table 1; 1:7,000 dilution, ab201060), 30 min at RT. After washing in TBST, membranes were incubated with secondary goat anti-rabbit HRP-conjugated IgG antibody (Table 1; 1:30,000 dilution, Abcam ab6721, RRID: AB_955,447). For negative control, membranes were incubated with secondary antibodies for 30 min at RT. Blots were washed in TBST and the chemiluminescent signal was visualized with the Clarity ECL Substrate (BioRad Laboratories, Hercules, CA, United States) and the Chemi Doc-It imaging system (UVP, Upland, CA, United States).

### 2.10. Statistical analysis

All data were analyzed for normality and appropriate tests were used. Student’s *t-*test or Mann–Whitney test was performed for the results of Elisa and biochemical analysis. All values are presented as mean ± SD as indicated in Figure legends and Supplementary Tables 1–6. The values of *p* < 0.05 were considered statistically significant. For all analysis and graphical presentation GraphPad Prism 9.0 (San Diego, CA) software package was used.

## 3. Results

To confirm STZ effects 54 days after administration and validate this animal model, we have compared the parameters of interest of the Control group with the STZ group. Since our experiment, as many studies before proved the specific toxicity of STZ, for purpose of simplicity and translatability, we have further tested only STZ + iTBS and the STZ + Placebo group. The exception was made for immunohistochemistry where we had to determine if astrogliosis was STZ-induced, so we included the Control group.

### 3.1. Streptozotocin induces oxidative stress and suppresses antioxidative capacity

**Figure 2:**
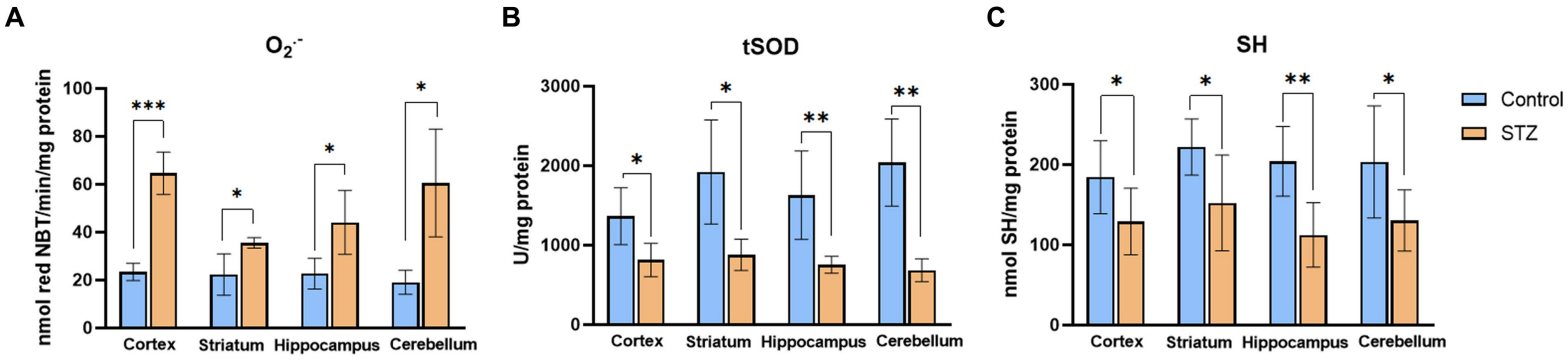
Streptozotocin induces oxidative stress and suppresses antioxidative defense. Evaluation of **(А)** Superoxide anion radical (O_2_^•−^; nmol red NBT/min/mg protein), **(B)** total Superoxide dismutase (tSOD; U/mg protein), and **(C)** Sulfhydryl groups (SH; nmol SH/mg protein) in the cortex, striatum, hippocampus, and cerebellum of the Wistar rats. The Control group received Saline solution intracerebroventricularly (icv) and Streptozotocin (STZ) group received icv streptozotocin (3 mg/kg). Bars in the graphs represent means ± SD values (unpaired *t*-test) for 6 animals in each group. ^*^p < 0.05, ^**^p < 0.01, ^***^p < 0.001.

The effects of STZ were evaluated by measuring levels of oxidative stress marker (O_2_^•−^), enzymatic and nonenzymatic components of antioxidative protection (tSOD, SH) in the Control and STZ group of selected brain structures (cortex, striatum, hippocampus, and cerebellum; Figure 2). Results showed that STZ increased oxidative stress and attenuated antioxidative capacity 54 days after STZ administration. Among all examined structures, the most noticeable change in O_2_^•−^ occurred in the cortex.

### 3.2. Intermittent theta burst stimulation attenuates STZ-induced oxidative and nitrosative stress in the cortex, striatum, hippocampus, and cerebellum

**Figure 3:**
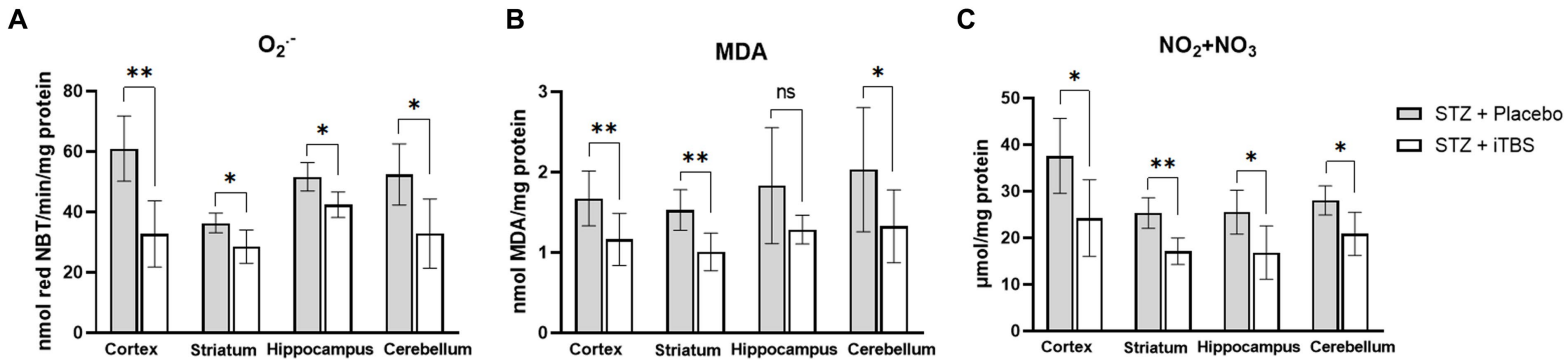
Intermittent Theta Burst Stimulation (iTBS) attenuates STZ-induced oxidative/nitrosative stress. Evaluation of **(A)** Superoxide anion radical (O_2_^•−^; nmol red NBT/min/mg protein), **(B)** malondialdehyde (MDA; mmol MDA/mg protein), and **(C)** nitrite and nitrate concentration (NO_2_ + NO_3_; μmol/mg protein) in the cortex, striatum, hippocampus, and cerebellum of the Wistar rats. The STZ + Placebo group represents STZ-administrated animals (3 mg/kg) subjected to noise artifact, and the STZ + iTBS group represents STZ-administrated rats (3 mg/kg) with applied iTBS protocol. Bars in the graphs represent means ± SD values (unpaired *t*-test) for 6 animals in each group. ^*^p < 0.05, ^**^p < 0.01, ^***^p < 0.001.

Superoxide anion and MDA levels were used as markers of oxidative stress, while NO_2_ + NO_3_ was used as a marker of nitrosative stress (Figure 3) in all examined brain structures. When compared with the STZ + Placebo group, iTBS stimulation (STZ + iTBS group) decreased O_2_^•−^ in all examined brain structures with the most noticeable change detected in the cortex. The iTBS decreased MDA levels in the cortex, striatum, and cerebellum, while in the hippocampus difference was not detected. The iTBS reduced levels of NO_2_ + NO_3_ in all examined structures, with the highest influence in the striatum.

### 3.3. Intermittent theta burst stimulation attenuates RNA and DNA damage in STZ-administrated rats

**Figure 4:**
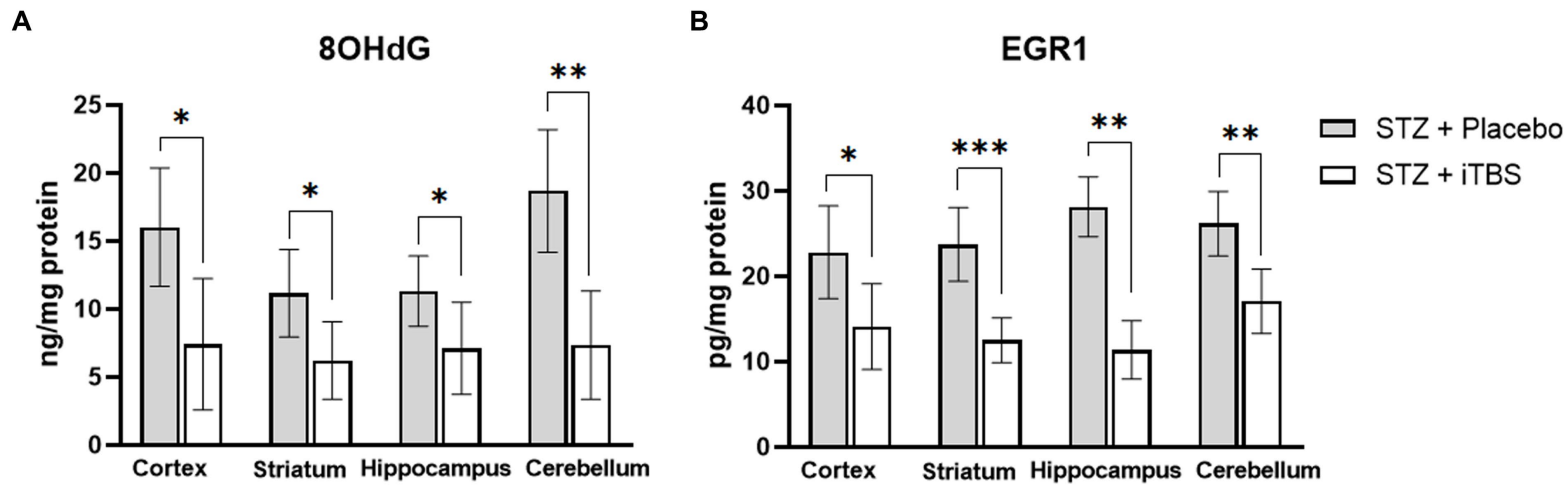
Intermittent Theta Burst Stimulation (iTBS) attenuates STZ-induced nucleic acid damage. Evaluation of 8-hydroxy-2′-deoxyguanosine **(A)** (8-OHdG; ng/mg protein) and Early growth response protein 1 **(B)** (EGR1; pg./mg protein) in the cortex, striatum, hippocampus, and cerebellum of the Wistar rats. The STZ + Placebo group represents STZ-administrated animals (3 mg/kg) subjected to noise artefict, and the STZ + iTBS group represents STZ-administrated rats (3 mg/kg) with applied iTBS protocol. Bars in the graphs represent means ± SD values (unpaired *t*-test and Mann–Whitney) for 6 animals in each group. ^*^p < 0.05, ^**^p < 0.01, ^***^p < 0.001.

Levels of 8-hydroxyguanosine (8OHdG) and Early Growth Response—1 (EGR1) were used as biomarkers of nucleic acid degradation, both partially caused by oxidative stress (Chen and Zhong, 2014). The iTBS significantly reduced both markers in all examined brain structures (Figure 4).

### 3.4. Intermittent theta burst stimulation decreases STZ-induced Aß deposits

**Figure 5:**
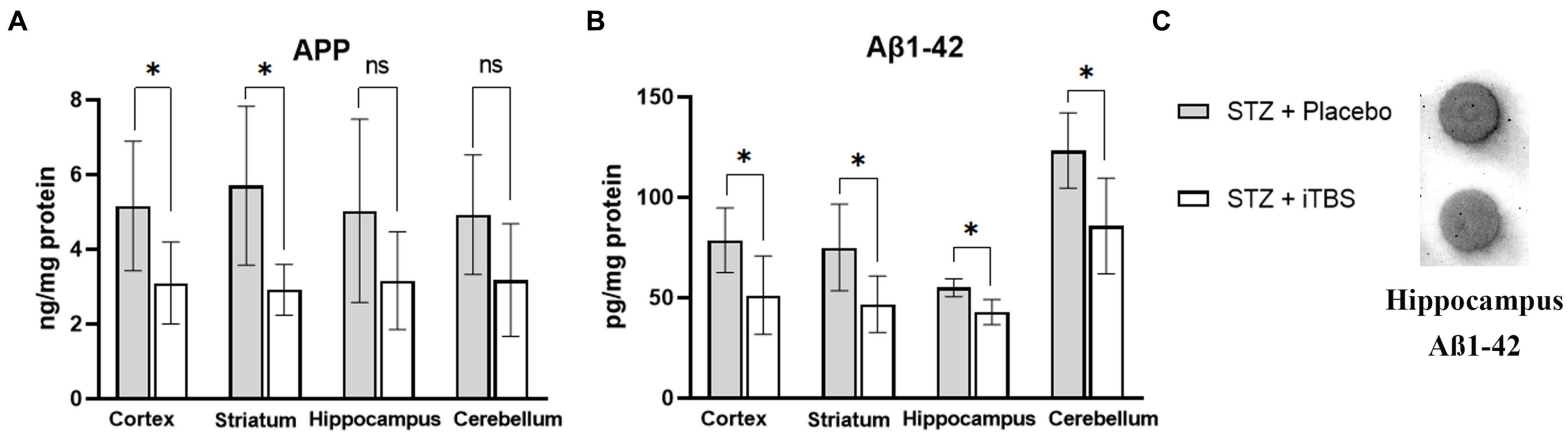
Intermittent Theta Burst Stimulation (iTBS) attenuates STZ-induced Aß deposition. Evaluation of **(A)** Amyloid ß precursor protein (APP; ng/mg protein) and **(B)** *ß*-amyloid_1-42_ (Aß_1-42_; pg./mg protein) in the cortex, striatum, hippocampus, and cerebellum of the Wistar rats. Evaluation of **(C)** *ß*-amyloid_1-42_ in the hippocampus using Dot Blot. The STZ + Placebo group represents STZ-administrated animals (3 mg/kg) subjected to noise artifact, and the STZ + iTBS group represents STZ-administrated rats (3 mg/kg) with applied iTBS protocol. Bars in the graphs represent means. ± SD values (unpaired *t*-test and Mann–Whitney) for 6 animals in each group. ^*^p < 0.05, ^**^p < 0.01, ^***^p < 0.001.

The amyloid precursor protein (APP) and Aβ-_1-42_ peptide were used as the detection markers of neurotoxic amyloid plaques in selected brain structures (Figure 5). The iTBS decreased APP levels in the cortex and striatum, and no significant change was detected in the hippocampus or cerebellum. Elisa’s kit result showed that iTBS decreased levels of Aβ-_1-42_ in all examined structures. On the other hand, dot blot evaluation of the Aβ-_1-42_ peptide showed no difference in the hippocampus. The inconsistency of the results from the dot blot and Elisa kit analyses probably occurred due to the sensitivity of these tests, and hence probably resulted in the difference in β-amyloid values.

### 3.5. Intermittent theta burst stimulation increases antioxidative capacity in STZ-administrated rats

**Figure 6:**
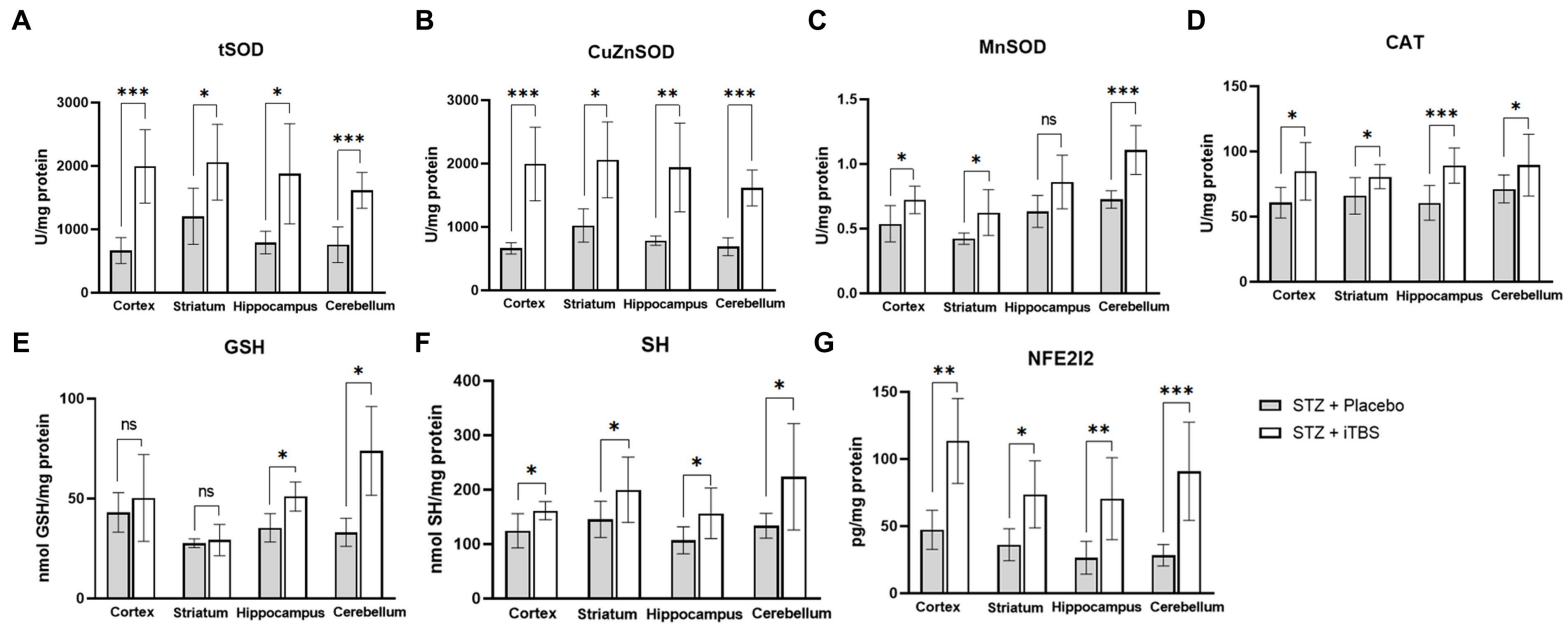
Intermittent Theta Burst Stimulation (iTBS) enhances antioxidative capacity in STZ-administrated rats. Evaluation of **(A)** total Superoxide dismutase (tSOD; U/mg protein), copper-zinc Superoxide dismutase **(B)** (CuZnSOD; U/mg protein), **(C)** manganese Superoxide dismutase (MnSOD; U/mg protein), **(D)** catalase (CAT; U/mg protein), **(E)** glutathione (GSH; nmol GSH/mg protein), **(F)** sulfhydryl groups (SH; nmol SH/mg protein), and **(G)** Nuclear factor erythroid-derived 2-like 2 (Nrf2; pg./mg protein) in the cortex, striatum, hippocampus, and cerebellum of the Wistar rats. The STZ + Placebo group represents STZ-administrated animals (3 mg/kg) subjected to noise artifact, and the STZ + iTBS group represents STZ-administrated rats (3 mg/kg) with applied iTBS protocol. Bars in the graphs represent means ± SD values (unpaired *t*-test and Mann–Whitney) for 6 animals in each group. ^*^p < 0.05, ^**^p < 0.01, ^***^p < 0.001.

The effects of iTBS on antioxidative capacity were evaluated through enzymatic (tSOD, CuZnSOD, MnSOD, CAT) and nonenzymatic (GSH, SH) components of an antioxidative system (Figure 6). The tSOD and CuZnSOD were increased in all examined structures, while the most observable changes were detected in the cortex and cerebellum in the STZ + iTBS group compared to the STZ + Placebo group. Also, MnSOD levels in the STZ + iTBS group were increased in the cortex and striatum compared to the STZ + Placebo group, and the highest impact was detected in the cerebellum, while the statistical significance was not detected in the hippocampus. The CAT activity was increased in all examined structures, while the highest change was detected in the hippocampus in STZ + iTBS group when compared to the STZ + Placebo group. The iTBS increased SH levels in all examined structures, while GSH levels were increased only in the hippocampus and cerebellum of STZ + iTBS group compared to the STZ + Placebo group. The iTBS significantly increased levels of Nrf2 in all examined structures (Figure 6) with the most notable change detected in the cerebellum.

### 3.6. Intermittent theta burst stimulation increases BDNF expression in STZ-administrated rats

**Figure 7:**
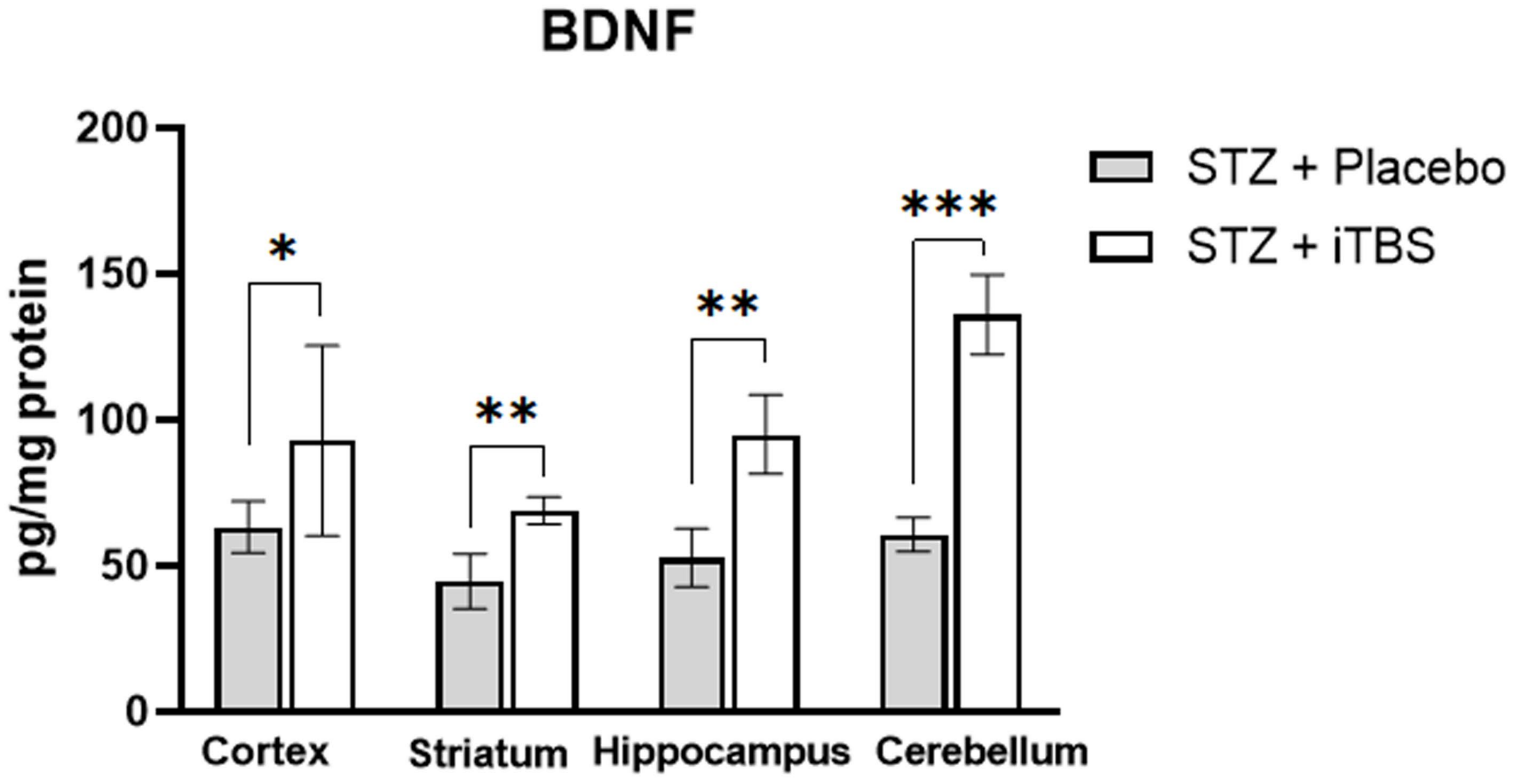
Intermittent Theta Burst Stimulation increases BDNF expression in STZ-administrated rats. Evaluation Brain-derived neurotrophic factor (BDNF; pg./mg protein) in the cortex, striatum, hippocampus, and cerebellum of the Wistar rats. The STZ + Placebo group represents STZ-administrated animals (3 mg/kg) subjected to noise artifact, and the STZ + iTBS group represents STZ-administrated rats (3 mg/kg) with applied iTBS protocol. Bars in the graphs represent means. ± SD values (unpaired *t*-test) for 6 animals in each group. ^*^p < 0.05, ^**^p < 0.01, ^***^p < 0.001.

The BDNF levels are elevated in all examined brain structures in STZ + iTBS group when compared to the STZ + Placebo group (Figure 7).

### 3.7. Intermittent theta burst stimulation attenuates STZ-induced reactive astrogliosis in the hippocampus

**Figure 8:**
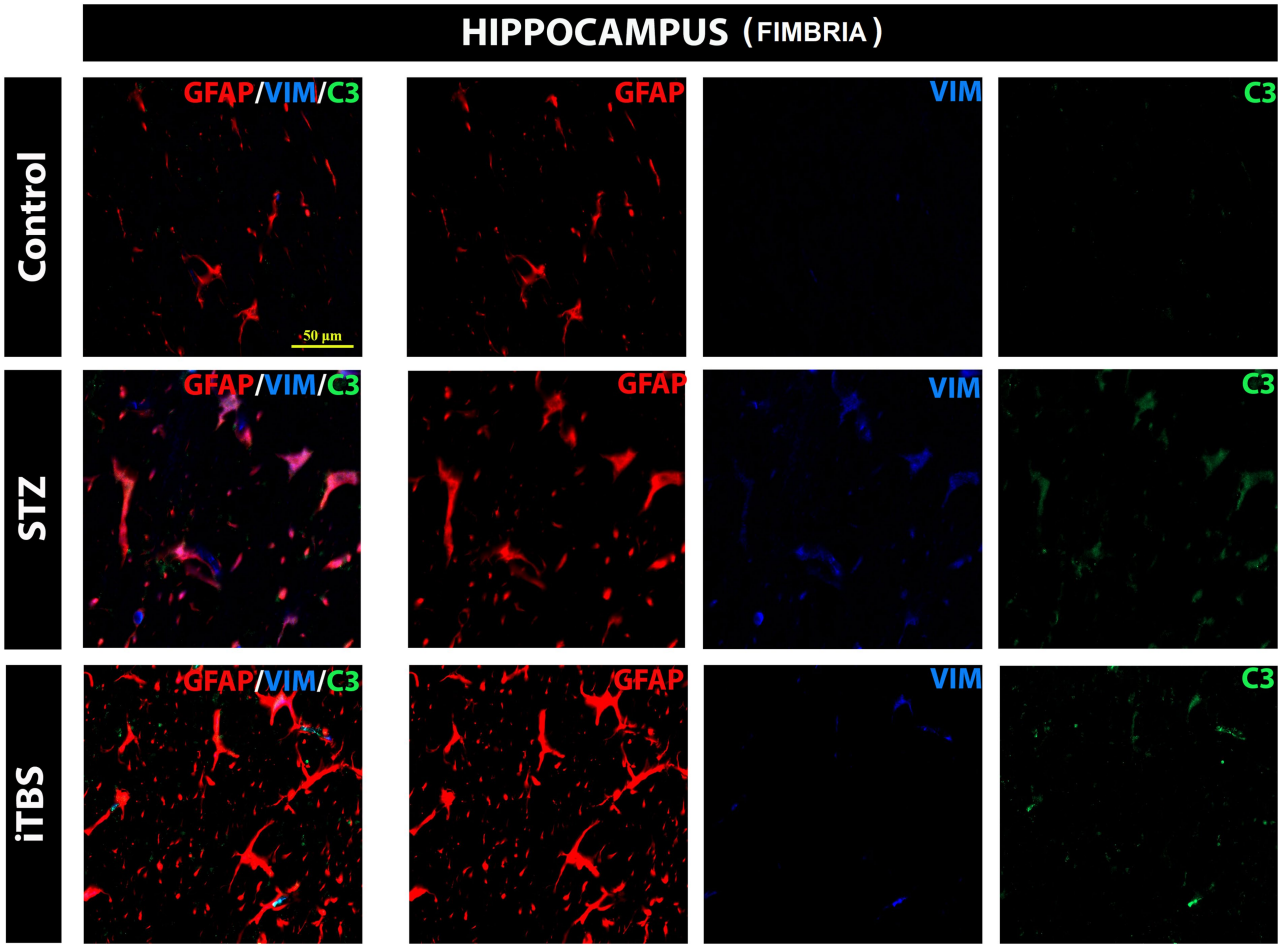
Effects of Theta Burst Stimulation on Streptozotocin induced reactive astrogliosis in the hippocampus (fimbria). Triple immunofluorescence labeling directed to astrocyte marker GFAP (red), vimentin—VIM (blue), and C3 (green). The number of tested rats was 3 for each group. The micrographs were taken at a magnification of 40X. The scale bar corresponds to 50 μm.

The most notable changes are usually detected in the hippocampus of AD patients and animal models and for this reason, it was chosen for the evaluation of astrocytes reactivity. The results showed normal distribution of GFAP-positive cells in the hippocampus (fimbria) of the Control group. On the other hand, in the STZ group, besides GFAP, VIM and C3 positive cells were detected with visible colocalization. In the iTBS group colocalization of GFAP/VIM/C3 was greatly attenuated, as well as the detection of VIM^+^ and C3^+^ cells. No reactive astrogliosis was detected in other regions examined, so it was not possible to determine the effects of iTBS on astrogliosis (Figure 8).

## 4. Discussion

In the present study, the effects of the iTBS protocol were investigated in the context of antioxidant, anti-inflammatory, and anti-amyloidogenic effects in different brain regions in the STZ-induced animal model of sAD-like pathology. Intracerebroventricular injection of STZ induces pathological changes at molecular, cellular, and behavioral levels—similar to those observed in the sporadic form of AD (Salkovic-Petrisic et al., 2013; Grieb, 2016). Upon central application, STZ generates central insulin resistance resulting in glucose hypometabolism, mitochondrial damage, and the generation of intracellular oxidative and nitrosative stress (Liu et al., 2022). It has been demonstrated that STZ promotes oxidative/nitrosative stress in rat brains detected as early as 1 week post-application and was present even 8 weeks following the acute application, suggesting ongoing degenerative processes (Sharma and Gupta, 2001; Ishrat et al., 2006; Pathan et al., 2006; Shoham et al., 2007; Saxena et al., 2011; Alluri et al., 2020; Akhtar et al., 2021). Some studies showed that icv STZ application alters the oxidative/nitrosative balance in several brain regions, including the hippocampus and cortex (Alluri et al., 2020; Akhtar et al., 2021), however, other brain regions were not examined in detail. To confirm the pro-oxidative status in our experimental conditions, we examined canonical markers of oxidative stress 8 weeks after STZ application. In this animal model, STZ increased the level of free O_2_^•−^ and reduced both enzymatic (tSOD activity) and nonenzymatic (SH groups) parameters of antioxidative capacity in all examined brain regions. These results confirm that icv STZ induces chronic degenerative changes coupled with oxidative imbalance, not only in the structures related to learning and memory processes, but in virtually all examined brain regions. It has been shown that STZ-induced oxidative stress can directly trigger lipid peroxidation resulting in increased levels of MDA (Farbood et al., 2020), as well as the change in the methylation pattern of DNA, leading to a cascade of neurodegenerative events marked by an increased 8OHdG level (Liu et al., 2022). On the other hand, antioxidative mechanisms have been shown to be significantly impaired in STZ-administrated rats, mostly noted in the cortex and hippocampus region (Alluri et al., 2020; Bavarsad et al., 2020; Akhtar et al., 2021; Liang et al., 2021; Tiwari et al., 2021).

Oxidative stress continues to be a key therapeutic target for neurological diseases. In developing antioxidant therapies for neurological disease, special attention should be given to the brain’s unique vulnerability of the brain to oxidative insults and its architecture (Birngruber et al., 2013). rTMS has been postulated as a promising therapeutic approach in the treatment of neurodegenerative disorders, through the production of complex neurobiological effects such as induction of early genes, changes in Ca^2+^ dynamics, reduction of oxidative stress and inflammation, and activation of neurotrophic factors (Uzair et al., 2022). Herein, we applied iTBS, a highly efficient protocol of rTMS, to evaluate its effect on oxidative stress and the promotion of antioxidative capacity. Our data demonstrated that iTBS treatment significantly reduced levels of oxidative stress markers with a concomitant increase in the antioxidative capacity (confirming previous data Stevanovic et al., 2020) in all brain regions, assessed through endogenous enzymatic components (CuZnSOD, MnSOD, SOD, CAT) and non-enzymatic components (GSH, SH) which are attenuated in STZ-induced neurotoxicity evaluated in other studies (Alluri et al., 2020; Bavarsad et al., 2020; Akhtar et al., 2021; Liang et al., 2021; Tiwari et al., 2021). The expression of antioxidant genes is partially regulated by the Nrf2 transcription factor. Under the conditions of increased production of reactive oxidative species, Nrf2 enters the nucleus and binds to antioxidant response elements (ARE; Ke et al., 2021), thus promoting the expression of antioxidant-related genes (SOD, GSH, CAT), which in turn may reduce the production of reactive oxygen species (Liang et al., 2021). Downregulation of Nrf2 has been reported in STZ-administrated animals (Liang et al., 2021) despite elevated markers of oxidative stress, indicating that Nrf2 was not translocating from the cytoplasm into the nucleus or that some processes may be blocking Nrf2 nuclear activity (Davies et al., 2021). One of the factors shown to initiate Nrf2 translocation is BDNF (Bruna et al., 2018; Davies et al., 2021), whose expression is proven to be modulated by rTMS (Stevanovic et al., 2019). Our results demonstrated TMS-mediated elevation of both Nrf2 and BDNF, which might partially explain the improvement of overall oxidative status (Numakawa et al., 2018; Liang et al., 2021; Uzair et al., 2022.) following STZ administration. Other studies carried out in rat models of Huntington’s disease (Túnez et al., 2006) and olfactory bulbectomy (Tasset et al., 2010) corroborate the antioxidant actions of rTMS, suggesting that it may be mediated through the Nrf2 pathway. Furthermore, it has been shown that rTMS may improve mitochondrial viability (Medina-Fernandez et al., 2017), which is a significant source of reactive oxygen species, and attenuate pro-apoptotic cascade improving cellular viability in general (Uzair et al., 2022). Interestingly, both BDNF and Nrf2 are involved in synaptic plasticity, learning, and memory which are impaired in STZ-induced neurotoxicity (Singh and Garabadu, 2021; Tiwari et al., 2021; Stanojevic et al., 2022). Given that we previously showed improvement in learning and memory after iTBS, these results might provide additional rationale for the observed improvement (Stanojevic et al., 2022). As a marker of cellular damage and death, we measured 8OHdG levels, which may correlate with degenerative disorders in nervous tissue. We found a marked decrease of 8OHdG levels following iTBS, which taken together with other data may indicate improved survival of the nerve cells. However, we should be careful before declaring iTBS attenuates DNA damage, as there is a normal turnover of nucleic acid degradation in every cell and 8OHdG level does not always indicate damage or death. At first, increased levels of DNA strand breaks in the AD model (Mehla et al., 2013; Kamat, 2015; Grieb, 2016) were considered to be part of apoptosis, but now it is widely accepted that oxidative damage is responsible for DNA strand breaks (Gella and Durany, 2009). Altogether, our results showed that iTBS reduced STZ-induced oxidative damage and restored the antioxidant capacity, indicating its excellent antioxidant action.

Next, we sought to explore the levels of APP and Aβ which are regarded as a hallmark of AD and AD-like models, and are shown to be significantly increase following STZ application (Salkovic-Petrisic et al., 2013; Alluri et al., 2020; Gáspár et al., 2021; Singh and Garabadu, 2021). Increased production of APP initiates various cellular signaling cascades, one of them being the transcription of the EGR1 gene (Hendrickx et al., 2013), which is markedly increased in the STZ-induced model of AD-like pathology (Alluri et al., 2020) and may regulate levels of Aβ (Qin et al., 2017). Furthermore, decreased expression of EGR1, as seen in this study following iTBS, can be linked to improvement in STZ-induced memory impairment (Stanojevic et al., 2022). A recent study suggested that impeded binding of EGR1 to the BACE1 promoter blocked the activation of the APP signaling, ultimately protecting neurons (He et al., 2022). Additionally, Aβ synthesis in the hippocampus is regulated by EGR1 (Qin et al., 2017), and EGR1 suppression has been shown to attenuate AD pathology (He et al., 2022). Simultaneously we registered attenuation of Aβ-_1-42_ in all examined structures after iTBS which wasn’t followed up with changes in Aβ-_1-42_ amount in the hippocampus after dot blot analysis. Indeed, Aβ levels are significantly increased in animal model of STZ-induced AD-like pathology (Salkovic-Petrisic et al., 2013; Alluri et al., 2020; Gáspár et al., 2021; Singh and Garabadu, 2021), but the exact time of the appearance of amyloid plaques after the toxin application is still debated and depends on many factors, such as the animal species, applied dose of toxin, and evaluation methodology. Thus, the noted variance in our results may come from the different sensitivity of these tests.

Finally, we wanted to examine astrocytes’ response to the iTBS treatment as they exert a major role in the regulation of oxidative stress in CNS (Chen et al., 2020), deeply involved in the development of AD pathology. Neurons exposed to significant oxidative stress strongly rely on antioxidant support from surrounding astrocytes which may employ antioxidative systems through the Nrf2-mediated pathway. On the other hand, under certain pathological conditions, astrocytes become one of the main sources of detrimental free radicals and directly promote neural damage (Chen et al., 2020). Astrocytes can directly or indirectly respond to electrical activity and, based on accumulating evidence, now it is strongly implicated that astrocytes are cellular effectors of TMS (Cullen and Young, 2016) and iTBS, respectively, (Dragic et al., 2020; Dragić et al., 2021; Stanojevic et al., 2022; Stekic et al., 2022). Our results showed STZ-induced GFAP/VIM/C3 colocalization in the hippocampus, which was a marker of reactive astrocytes (Dragić et al., 2019), specifically in fimbria, which was adjacent to lateral ventricles where the toxin had been applied, indicating reactive astrogliosis persists even 8 weeks after STZ administration. Furthermore, iTBS significantly reduced the number of VIM^+^ and C3^+^ cells in the hippocampus. This is strong evidence that iTBS reduces reactive gliosis in the hippocampus, which is in accordance with our previous results (Stanojevic et al., 2022) in preventing its detrimental role. Most of the tested parameters were observed in all examined structures, which might point to STZ-induced regional-nonspecific effects. Given that STZ is applied icv., and that both lateral ventricles communicate with the 3rd and 4th ventricles, toxin could diffuse practically through the whole brain (Salkovic-Petrisic et al., 2013). Accordingly, iTBS has showed improvement in all parameters which may be due to non-focal stimulation, but rather whole brain stimulation as the size of the coil does not allow focal application of the magnetic stimulation.

Based on the results acquired so far, iTBS represents an excellent candidate for further research as an add-on therapy for AD, as it exerts antioxidative, anti-amyloidogenic, and anti-inflammatory properties in the animal model of STZ-induced sAD-like pathology. However, we should point out some weaknesses of our study. Limited by the coil size, an accurate area of stimulation cannot be achieved on the rat (Mancic et al., 2016). So, our results reflect the effects of two sets of five-days long iTBS stimulation on subcortical and cortical structures and their interconnections. Numerous factors besides the coil size and shape, such as the site of stimulation, frequency, intensity, and the number of runs affect the induction of long-term changes in cortical and subcortical excitability, and further investigation should be pointed to a more focal stimulation. Special attention should be paid to STZ icv dose- and time-dependent patterns of pathophysiology in order to provide better predictive value in translating the results to humans.

## 5. Conclusion

This study has confirmed that STZ-induced AD-like pathology leads to the induction of oxidative/ nitrosative stress accompanied by reduced antioxidative protection, as well as reactive astrogliosis, and pathological amyloidogenesis. The iTBS therapy has been proposed to have a beneficial role in neurodegenerative disorders due to its neuroprotective antioxidant, anti-inflammatory and anti-amyloidogenic effects.

## Data availability statement

The original contributions presented in the study are included in the article/Supplementary material, further inquiries can be directed to the corresponding author.

## Ethics statement

The animal study was reviewed and approved by Ethics Committee for Animal Welfare and the Ministry of Agriculture, forestry, and water economy of the Republic of Serbia, decision no. 323-07-08358/2020-05.

## Author contributions

ISte, MN, ISto, and TI contributed to the conception and design of the study. JS, ISte, MN, MZ, and MD conducted a research and investigation process and performed the experiments and data/evidence collection. JS, ISte, and MD performed statistical analysis and wrote the first draft of the manuscript. All authors contributed to the article and approved the submitted version.

## Funding

This work was financially supported by the University of Defense (grant no: MFVMA/02/22-24) and by the Ministry of science technological development and innovation of the Republic of Serbia (grant no. 451-03-47/2023-01/200113).

## Conflict of interest

The authors declare that the research was conducted in the absence of any commercial or financial relationships that could be construed as a potential conflict of interest.

## Publisher’s note

All claims expressed in this article are solely those of the authors and do not necessarily represent those of their affiliated organizations, or those of the publisher, the editors and the reviewers. Any product that may be evaluated in this article, or claim that may be made by its manufacturer, is not guaranteed or endorsed by the publisher.
