# Supplementary material for: Intermittent theta burst stimulation attenuates oxidative stress and reactive astrogliosis in the streptozotocin-induced model of Alzheimer’s disease-like pathology
Source: Front Aging Neurosci. 2023 May 18;15:1161678. doi: 10.3389/fnagi.2023.1161678 (PMC10233102; doi:10.3389/fnagi.2023.1161678)
Supplement: Supplementary file 1 [file Data_Sheet_1.docx]

**Supplementary**

SP1. Results of Unpaired t-test

| **PARAMETER** | | **GROUP**  (Mean value ± SD) | | **t value** | **p value** |
| --- | --- | --- | --- | --- | --- |
|  |  | **Control** | **STZ** |  |  |
| **O_2_**^•−^  (nmol red NBT/min/ mg protein) | Cortex | 23.43 ± 3.66 | 64.59 ± 8.83 | 8.618 | 0.0001 |
|  | Striatum | 22.34 ± 8.66 | 35.53 ± 2.15 | 2.958 | 0.0253 |
|  | Hippocampus | 22.69 ± 6.37 | 44.12 ±13.33 | 2.900 | 0.0273 |
|  | Cerebellum | 19.02 ± 5.01 | 60.51 ± 22.50 | 3.599 | 0,0114 |
| **tSOD**  (U/mg protein) | Cortex | 1363 ± 357 | 812.9 ± 209 | 2.903 | 0.0229 |
|  | Striatum | 1921 ± 656 | 878.8 ± 197 | 3.044 | 0.0227 |
|  | Hippocampus | 1630 ± 556 | 752.4 ± 106 | 3.512 | 0.0098 |
|  | Cerebellum | 2039 ± 549 | 684.7 ± 144 | 5.378 | 0.0010 |
| **SH**  (nmol SH/mg protein) | Cortex | 184.4 ± 45.6 | 129.2 ± 41.5 | 2.287 | 0.0430 |
|  | Striatum | 221.9 ± 35.1 | 152.3 ± 59.7 | 2.663 | 0.0207 |
|  | Hippocampus | 204.1 ± 43.5 | 112.4 ± 40.1 | 3.945 | 0.0023 |
|  | Cerebellum | 203.4 ± 69.9 | 130.3 ± 38.3 | 2.428 | 0.0318 |

SP2. Results of Unpaired t-test

| **PARAMETER** | | **GROUP**  (Mean value) | | **t value** | **p value** |
| --- | --- | --- | --- | --- | --- |
|  |  | **STZ + Placebo** | **STZ + iTBS** |  |  |
| **O_2_**^•−^  (nmol red NBT/ min/ mg protein) | Cortex | 60.98 ± 10.79 | 32.73 ± 10.96 | 4.031 | 0.0038 |
|  | Striatum | 36.33 ± 3.24 | 28.51 ± 5.51 | 2.859 | 0.0212 |
|  | Hippocampus | 51.65 ± 4.70 | 42.35 ± 4.19 | 3.089 | 0.0176 |
|  | Cerebellum | 52.46 ± 10.13 | 32.83 ± 11.45 | 2.567 | 0.0425 |
| **MDA**  (mmol MDA/mg protein) | Cortex | 1.67 ± 0.61 | 1.16 ± 0.32 | 3.156 | 0.0065 |
|  | Striatum | 1.53 ± 0.25 | 1.01 ± 0.23 | 3.515 | 0.0066 |
|  | Hippocampus | 1.83 ± 0.72 | 1.29 ± 0.18 | 1.637 | 0.1298 |
|  | Cerebellum | 2.03 ± 0.77 | 1.33 ± 0.46 | 2.190 | 0.0474 |
| **NO_2_+NO_3_**  (μmol/mg protein) | Cortex | 37.57 ± 8.06 | 24.22 ± 10.11 | 2.836 | 0.0177 |
|  | Striatum | 25.30 ± 10.12 | 17.13 ± 4.15 | 4.408 | 0.0017 |
|  | Hippocampus | 25.52 ± 8.01 | 16.84 ± 6.26 | 2.613 | 0.0310 |
|  | Cerebellum | 28.01 ± 8.15 | 20.85 ± 6.06 | 2.932 | 0.0167 |

SP3. Results of Unpaired t-test and Mann-Whitney test

| **PARAMETER** | | **GROUP**  (Mean value) | | **t value** | **p value** |
| --- | --- | --- | --- | --- | --- |
|  |  | **STZ + Placebo** | **STZ + iTBS** |  |  |
| **8OHdG**  (ng/mg protein) | Cortex | 16.03 ± 4.34 | 7.41 ± 4.84 | 3.114 | 0.0124 |
|  | Striatum | 11.16 ± 3.21 | 6.21 ± 2.86 | 2.667 | 0.0257 |
|  | Hippocampus | 11.32 ± 2.57 | 7.11 ± 3.39 | 2.345 | 0.0437 |
|  | Cerebellum | 18.69 ± 4.51 | 7.35 ± 3.98 | 4.500 | 0.0011 |
| **EGR1**  (pg/mg protein) | Cortex | 22.82 ± 5.43 | 14.09 ± 5.02 | 2.827 | 0.0179 |
|  | Striatum | 23.73 ± 4.31 | 12.50 ± 2.60 | 5.151 | 0.0004 |
|  | Hippocampus | 28.15 ± 3.48 | 11.36 ± 3.41 | 0 Mann-Whitney | 0.0025 |
|  | Cerebellum | 26.17 ± 3.79 | 17.09 ± 3.76 | 4.107 | 0.0021 |

SP4. Results of Unpaired t-test and Mann-Whitney test

| **PARAMETER** | | **GROUP**  (Mean value) | | **t value** | **p value** |
| --- | --- | --- | --- | --- | --- |
|  |  | **STZ + Placebo** | **STZ + iTBS** |  |  |
| **APP**  (ng/mg protein) | Cortex | 5.16 ± 1.73 | 3.31 ± 1.1 | 2 Mann-Whitney | 0.0173 |
|  | Striatum | 5.70 ± 2.12 | 2.91 ± 0.68 | 2.798 | 0.0189 |
|  | Hippocampus | 5.03 ± 2.45 | 3.28 ± 1.01 | 10 Mann-Whitney | 0.2677 |
|  | Cerebellum | 4.93 ± 1.60 | 3.18 ± 1.51 | 1.784 | 0.1122 |
| **Aß**  (pg/mg protein) | Cortex | 78.63 ± 16.02 | 51.23 ± 19.58 | 2.670 | 0.0235 |
|  | Striatum | 74.98 ± 21.53 | 46.70 ± 14.07 | 2.554 | 0.0287 |
|  | Hippocampus | 55.01 ± 4.48 | 42.79 ± 6.28 | 3.032 | 0.0290 |
|  | Cerebellum | 123.4 ± 18.74 | 85.73 ± 23.79 | 2.779 | 0.0239 |

SP5. Results of Unpaired t-test and Mann-Whitney test

| **PARAMETER** | | **GROUP**  (Mean value) | | **t value** | **p value** |
| --- | --- | --- | --- | --- | --- |
|  |  | **STZ + Placebo** | **STZ + iTBS** |  |  |
| **tSOD**  (U/mg protein) | Cortex | 665.2 ± 203.5 | 1994 ± 579.6 | 5.258 | 0.0005 |
|  | Striatum | 1204 ± 441.5 | 2060 ± 597.4 | 2.734 | 0.0231 |
|  | Hippocampus | 790.5 ± 177.1 | 1876 ± 790.8 | 3.337 | 0.0103 |
|  | Cerebellum | 757.7 ± 281.1 | 1615 ± 283 | 5.022 | 0.0007 |
| **CuZnSOD**  (U/mg protein) | Cortex | 662.6 ± 88.2 | 1993 ± 579.7 | 5.073 | 0.0010 |
|  | Striatum | 1022 ± 262.2 | 2059 ± 597.6 | 3.119 | 0.0151 |
|  | Hippocampus | 782.9 ± 72.6 | 1939 ± 699.6 | 3.675 | 0.0063 |
|  | Cerebellum | 687.8 ± 140.1 | 1614 ± 283.1 | 6.556 | 0.0002 |
| **MnSOD**  (U/mg protein) | Cortex | 0.54 ± 0.14 | 0.72 ± 0.11 | 2.462 | 0.0336 |
|  | Striatum | 0.42 ± 0.04 | 0.62 ± 0.18 | 2.470 | 0.0387 |
|  | Hippocampus | 0.63 ± 0.12 | 0.86 ± 0.21 | 2.253 | 0.0508 |
|  | Cerebellum | 0.73 ± 0.11 | 1.11 ± 0.19 | 5.010 | 0.0005 |
| **CAT**  (U/mg protein) | Cortex | 60.70 ± 11.72 | 84.71 ± 20.43 | 2.813 | 0.0138 |
|  | Striatum | 65.95 ± 13.98 | 80.58 ± 9.19 | 2.544 | 0.0217 |
|  | Hippocampus | 60.55 ± 13.41 | 89.17 ± 13.52 | 4.374 | 0.0005 |
|  | Cerebellum | 71.23 ± 10.66 | 89.49 ± 23.65 | 2.191 | 0.0436 |
| **GSH**  (nmol GSH/mg protein) | Cortex | 43.08 ± 9.91 | 50.23 ± 21.70 | 0.663 | 0.5283 |
|  | Striatum | 27.63 ± 2.17 | 29.21 ± 7.82 | 0.390 | 0.7097 |
|  | Hippocampus | 35.33 ± 7.07 | 51.00 ± 7.31 | 3.083 | 0.0216 |
|  | Cerebellum | 33.08 ± 7.04 | 73.86 ± 22.21 | 3.501 | 0.0128 |
| **SH**  (nmol SH/mg protein) | Cortex | 124.4 ± 31.36 | 161.4 ± 16.42 | 2.615 | 0.0226 |
|  | Striatum | 145.4 ± 33.38 | 199.8 ± 59.98 | 2.269 | 0.0409 |
|  | Hippocampus | 107.1 ± 24.76 | 156.7 ± 73.20 | 2.537 | 0.0276 |
|  | Cerebellum | 133.4 ± 22.64 | 223.7 ± 97.83 | 2.543 | 0.0234 |
| **NFE2L2**  (pg/mg protein) | Cortex | 47.76 ± 14.51 | 113.4 ± 31.65 | 0 Mann-Whitney | 0.0022 |
|  | Striatum | 36.04 ± 16.61 | 73.57 ± 25.03 | 3.057 | 0.0136 |
|  | Hippocampus | 26.45 ± 12.19 | 70.38 ± 30.54 | 3.553 | 0.0035 |
|  | Cerebellum | 28.33 ±8.04 | 90.82 ± 36.61 | 4.411 | 0.0008 |

SP6. Results of Unpaired t-test

| **PARAMETER** | | **GROUP**  (Mean value) | | **t value** | **p value** |
| --- | --- | --- | --- | --- | --- |
|  |  | **STZ + Placebo** | **STZ + iTBS** |  |  |
| **BDNF**  (pg/mg protein) | Cortex | 63.27 ± 8.86 | 92.84 ± 32.55 | 2.327 | 0.0423 |
|  | Striatum | 44.64 ± 9.38 | 68.96 ± 4.63 | 4.101 | 0.0064 |
|  | Hippocampus | 52.74 ± 9.84 | 94.95 ± 13.58 | 5.116 | 0.0022 |
|  | Cerebellum | 60.58 ± 5.81 | 136.1 ± 13.67 | 10.140 | 0.0002 |
